# Ursolic Acid Regulates Immune Balance, Modulates Gut Microbial Metabolism, and Improves Liver Health in Mice

**DOI:** 10.3390/ijms251910623

**Published:** 2024-10-02

**Authors:** Man Zhao, Yali Cui, Fengxia Wang, Fengyang Wu, Chong Li, Shudong Liu, Baojiang Chen

**Affiliations:** 1 College of Animal Science and Technology, Hebei Agricultural University, Baoding 071051, China; 2 College of Traditional Chinese Veterinary Medicine, Hebei Agricultural University, Baoding 071051, China

**Keywords:** ursolic acid, gut health, liver protection, gut microbiota, metabolism, liver transcriptome

## Abstract

Ursolic acid (UA) has demonstrated significant immunomodulatory and hepatoprotective effects; however, the underlying mechanisms remain unclear. This study aims to analyze the impact of UA on the gut microbiome, metabolome, and liver transcriptome, investigate UA’s role in maintaining gut immune homeostasis and liver health, and evaluate the potential contributions of gut microbes and their metabolites to these beneficial effects. Our findings indicate that UA enhances immune balance in the jejunum, fortifies intestinal barrier function, and promotes overall gut health. UA modulates the intestinal microbiota and its metabolic processes, notably increasing the abundance of beneficial bacteria such as *Odoribacter* and *Parabacteroides*, along with their metabolites, including ornithine and lactucin. Additionally, UA inhibits the expression of interleukin-1 receptor 1 (*IL1R1*) and calcium (Ca^2+^) voltage-gated channel auxiliary subunit beta 2 (*CACNB2*) while enhancing the synthesis pathways of retinol and ascorbic acid, thereby exerting a protective influence on liver function. In summary, UA enhances intestinal immune homeostasis and promotes liver health, with these advantageous effects potentially mediated by beneficial bacteria (*Odoribacter* and *Parabacteroides*) and their metabolites (ornithine and lactucin).

## 1. Introduction

Body health is closely related to gut health, relying on moderate immunity, an intact gut barrier, and a stable gut microbe. The gut harbors a diverse array of immune cells, including T helper (Th) cells and T regulatory (Treg) cells, which work together to maintain the balance of the body’s immune response [1]. An overproduction of immune-related cytokines, such as interleukin (IL)-6, IL-1*β*, and IL-17A, can lead to intestinal cell damage and compromise the integrity of the gut barrier, consequently triggering systemic inflammation. In contrast, cytokines such as IL-10 and transforming growth factor-*β* (TGF-*β*) play a role in inhibiting inflammation [1]. Ursolic acid (UA) is a naturally occurring pentacyclic triterpene carboxylic acid found in various plants, either in free acid form or as an aglycone of triterpene saponins [2]. In recent years, there has been a surge of interest in UA, primarily concerning its pharmacological properties, which include antioxidant [3], antitumor [4], anti-inflammatory [5], and antimicrobial [2] effects. Our previous meta-analysis confirmed that UA can reduce levels of pro-inflammatory cytokines like IL-1*β* and IL-6 [6]. Furthermore, UA has been shown to activate the immune system and enhance the proliferation of immune cells [7]. Nevertheless, the regulatory mechanisms by which UA influences intestinal immune homeostasis and gut health remain unclear.

The gut microbiota plays a critical role in maintaining intestinal immune homeostasis and barrier function [8,9,10]. Research has demonstrated that gut microbes can modulate immune equilibrium through the regulation of Th17/Treg cell dynamics. For example, *Lactobacillus rhamnosus GG* is capable of modulating Th17/Treg homeostasis, thus aiding in the preservation of host intestinal health [11]. Similarly, *Bacteroides fragilis* enhances Treg cell activation by increasing *Foxp3* expression [12]. UA has exhibited significant regulatory effects on the gut microbiota [13,14,15]. Consequently, understanding how UA influences immune balance and gut health via the modulation of gut microbes presents a compelling area of investigation. Additionally, UA has been shown to mitigate liver damage by modulating enterohepatic axis homeostasis [13] and is recognized as a contributing factor to the hepatoprotective properties of various foods and medicinal plants [13,16]. The gut microbiota, a key component of the gut–liver axis (GLA), is also closely associated with liver health [8]. Studies indicate that *Lactobacillus* and *Bifidobacterium* can enhance metabolic processes in rats through the GLA [17]. However, the specific mechanisms through which UA exerts protective effects on the liver necessitate further exploration.

To address these questions, this study utilizes a multi-omics approach to analyze the effects of UA on the intestinal microbiota, metabolome, and liver transcriptome in mice. We evaluate the role of UA in immune balance and liver protection, elucidating the potential contributions of gut microbes and their metabolites to these processes.

## 2. Results

### 2.1. Effect of UA on Immune-Related Cytokines in Mice

**Figure 1:**
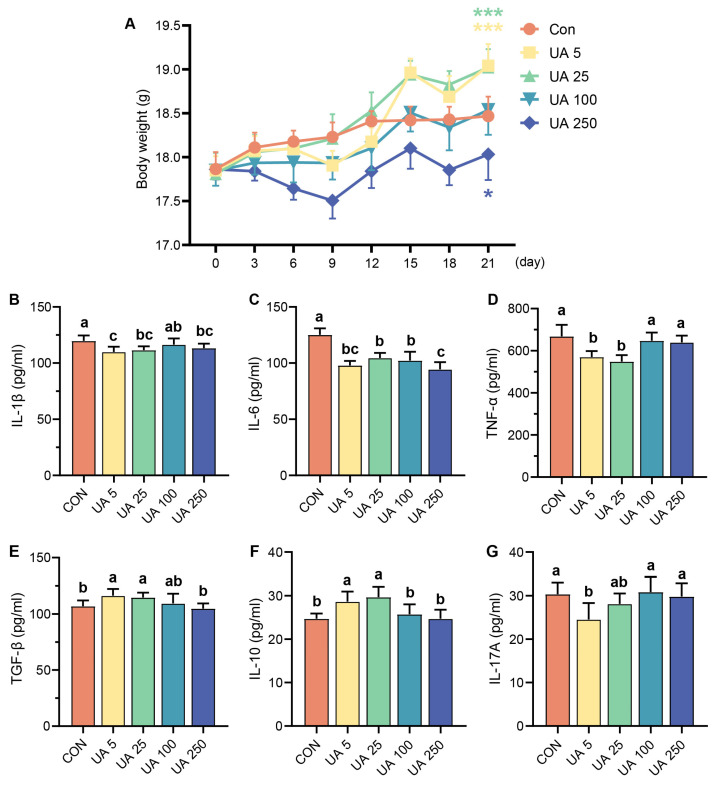
Effects of ursolic acid on body weight and immune-related cytokines in mice. (**A**) Body weight (*, *p* < 0.05, and ***, *p* < 0.001). (**B**) IL-1*β*, (**C**) IL-6, (**D**) TNF-*α*, (**E**) TGF-*β*, (**F**) IL-10, and (**G**) IL-17A cytokine levels in serum. Values with different letters (a, b, and c) differed significantly within a mice trial (*p* < 0.05). Data are shown as mean ± SD. *n* = 6.

As shown in Figure 1A, the body weight (BW) of mice in the UA 5 and UA 25 groups was significantly higher than that of the CON group (*p* < 0.05). Compared to the CON group, the UA 5 and UA 25 groups had significantly lower levels of interleukin (IL)-1*β*, IL-6, and tumor necrosis factor (TNF)-*α* (*p* < 0.05) and higher levels of transforming growth factor (TGF)-*β* and IL-10 (*p* < 0.05) (Figure 1B–G).

### 2.2. UA Promotes Intestinal Development, Intestinal Epithelial Barrier Integrity, and Immune Balance in Mice

**Figure 2:**
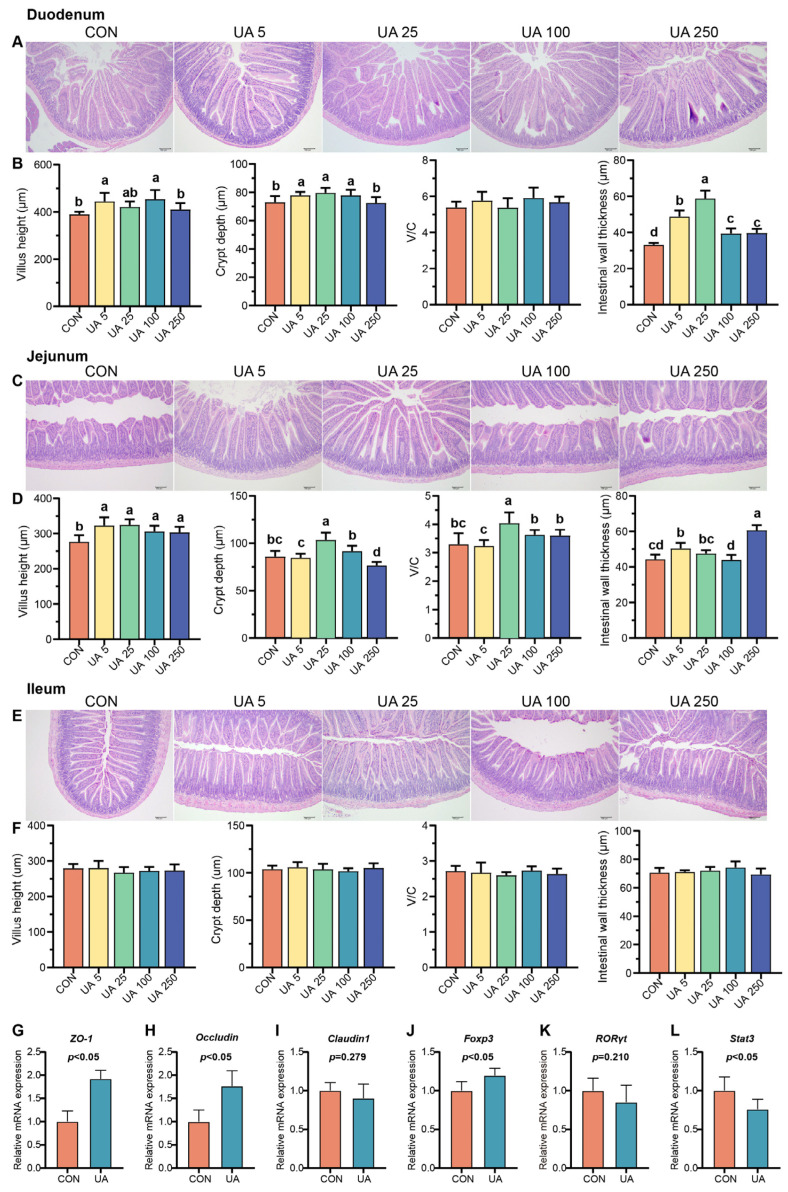
Effect of ursolic acid on the intestinal health of mice. Effect of ursolic acid on the intestinal morphology of the duodenum (**A**,**B**), jejunum (**C**,**D**), and ileum (**E**,**F**) in mice. Effects of ursolic acid on the expression of genes related to the intestinal barrier (**G**–**I**) and Th17/Treg cell-related factor (**J**–**L**) in the jejunum of mice. Values with different letters (a, b, c, and d) differed significantly within a mice trial (*p* < 0.05). Data are shown as mean ± SD. *n* = 6. Scale bars in (**A**,**C**,**E**) = 100 μm.

Mouse intestinal tissues (duodenum, jejunum, and ileum) were stained with H&E to explore the effect of UA on intestinal development in mice (Figure 2A–F). The results indicated that UA treatment (UA 5 and UA 100) significantly increased duodenal villus length (V), while (UA 5, UA 25, and UA 100) significantly increased duodenal crypt depth (C), (UA 5, UA 25, UA 100, and UA 250) significantly increased duodenal wall thickness (*p* < 0.05), and (UA 5, UA 25, UA 100, and UA 250) significantly increased duodenal crypt depth. However, there was no significant effect on the V/C ratio (*p* > 0.05). Compared to the CON group, UA treatment (UA 5, UA 25, UA 100, and UA 250) significantly increased jejunal villus length, (UA 25) crypt depth, and (UA 25) V/C and (UA 5 and UA 250) intestinal wall thickness (*p* < 0.05). UA treatment had no significant effect on the intestinal morphology (villus length, crypt depth, V/C, and intestinal wall thickness) of the ileum (*p* > 0.05). It is concluded that the effect of UA on the small intestine is mainly concentrated in the jejunum, with the optimal dose being the UA 25 group. Based on this, we further investigated the effects of optimal UA treatment (UA 25) on the intestinal barrier and immune balance in mice. The results showed that UA treatment significantly increased the mRNA expression of zonula occludens-1 (*ZO-1*) and *Occludin* in the jejunum of mice (*p* < 0.05) but had no significant effect on the mRNA expression of *Claudin1* (*p* > 0.05) (Figure 2G–I). Compared with the CON group, the UA-treated group had significantly higher forkhead box P3 (*Foxp3*) mRNA expression levels and significantly lower signal transducer and activator of transcription 3 (*Stat3*) mRNA expression levels (*p* < 0.05). UA had no significant effect on retinoid-related orphan receptor gamma t (*RORγt*) mRNA expression (*p* > 0.05) (Figure 2J–L) based on the optimal effects of the UA 25 group on body weight, serum immune factor levels, and intestinal tissue morphology, as well as beneficial effects on genes related to the intestinal barrier and immune balance. We selected the UA 25 group (UA group) for the omics study.

### 2.3. UA Changes the Composition and Structure of the Gut Microbiota in Mice

**Figure 3:**
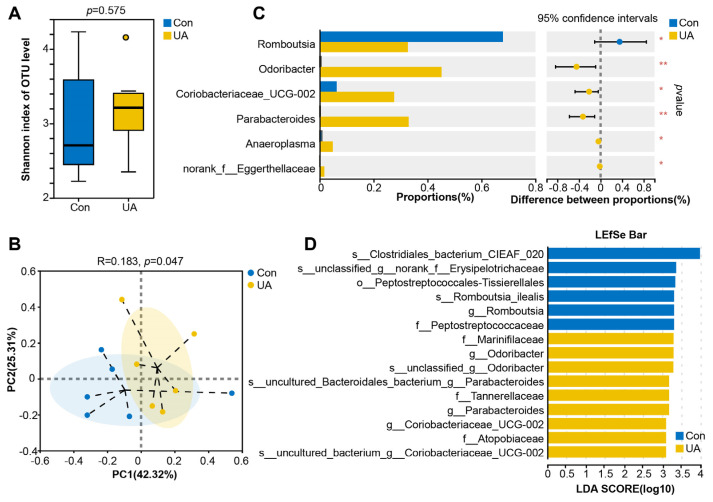
Effect of ursolic acid on gut microbiota in mice. (**A**) The comparison of microbiota diversity (Shannon index) and the Adonis analysis. Boxes denote the interquartile (IQR) between the first and third quartiles (25th and 75th percentiles, respectively) and the line inside denotes the median. (**B**) Principal coordinate analysis (PCoA) based on Bray–Curtis distance. (**C**) Comparison of differences in the relative abundance of bacteria at the genus level (*, *p* < 0.05, and **, *p* < 0.01). (**D**) Differentiating bacteria identified by LEfSe with LDA effect size ≥ 3. *n* = 6.

To explore whether the physiological effects of UA were related to the gut microbiota, we further investigated the effects of UA on the gut microbiota. Figure S1A–C illustrates the rank abundance curves, rarefaction curves, and Shannon curves derived from this gene sequencing. There was no significant difference in the Shannon index between the two groups (*p* > 0.05) (Figure 3A). The results of principal coordinate analysis (PCoA) indicated that the first principal component explained 42.32% of the variance between groups, and the second principal component explained 25.31% of the variance between groups. The results of the Adonis analysis demonstrated that UA treatment significantly regulated the structure of intestinal flora in mice (*p* < 0.05) (Figure 3B). Figure S1D,E presents the top 10 bacteria with relative abundance at the phylum level and the top 15 bacteria with relative abundance at the genus level. UA significantly reduced the relative abundance of *Romboutsia* and significantly increased the relative abundance of *Odoribacter*, *Coriobacteriaceae_UCG-002*, *Parabacteroides*, *Anaeroplasma*, and *Norank_f__Eggerthellaceae* (*p* < 0.05) (Figure 3C). Linear discriminant analysis (LDA) effect size (LEfSe) was employed to identify the core taxa (LDA ≥ 3, *p* < 0.05) that were most likely to explain the differences between groups. The results revealed that there were six taxa-marker bacteria in the CON group and nine taxa-marker bacteria in the UA group (Figure 3D and Figure S1F). Notably, *Romboutsia* was further identified as the marker bacteria in the CON group, while *Odoribacter*, *Coriobacteriaceae_UCG-002*, and *Parabacteroides* were identified as the marker bacteria in the UA group.

### 2.4. UA Also Altered Metabolism of Gut Microbiota

**Figure 4:**
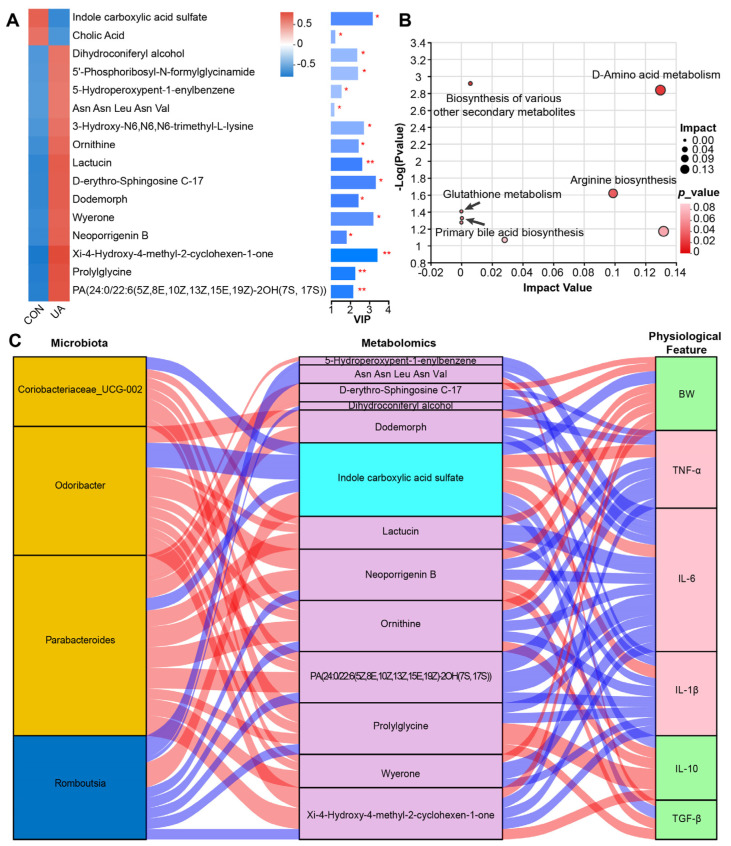
Effect of ursolic acid on the fecal metabolism of mice. (**A**) Heatmap of 16 different metabolites. The right side shows the variable importance in the projection (VIP) bar graph of the metabolite. The color of the bars indicates the significant difference in metabolites between the two groups of samples, and the smaller the *p*-value, the darker the color (*, *p* < 0.05; **, *p* < 0.01). (**B**) KEGG pathway enrichment analysis of differential metabolites. (**C**) Interrelationship between microbiota, metabolomics, and physiological features. Red connections indicate a positive correlation (Spearman correlation test, |R| > 0.6, *p* < 0.05,), while blue connections show correlations that were negative (Spearman correlation test, |R| > 0.6, *p* < 0.05). In the microbiota column, the orange stratum represents bacteria that were highly enriched in the UA group, and the stratum-colored blue was increased in the control group. In the metabolomics column, the purple stratum represents metabolites that were highly enriched in the UA group, and the stratum-colored ice blue was increased in the control group. In the physiological feature column, the green stratum represents bacteria that were highly enriched in the UA group, and the pink stratum was increased in the control group. *n* = 6.

A total of 355 metabolites were identified in the mouse fecal metabolome. Partial least squares discriminant analysis (PLS-DA) indicated that the mice in the CON group and the UA group exhibited different metabolic characteristics (Figure S2A,B). Sixteen metabolites were significantly different between the two groups, among which indole carboxylic acid sulfate and cholic acid were significantly enriched in the CON group. Dihydroconiferyl alcohol, 5′-phosphoribosyl-N-formylglycinamide, 5-hydroperoxypent-1-enylbenzene, Asn Asn Leu Asn Val, 3-hydroxy-*N*^6^,*N*^6^,*N*^6^-trimethyl-L-lysine, ornithine, lactucin, D-erythro-sphingosine C-17, dodemorph, wyerone, neoporrigenin B, Xi-4-hydroxy-4-methyl-2-cyclohexen-1-one, prolylglycine, and PA(24:0/22:6(5Z,8E,10Z,13Z,15E,19Z)-2OH(7S, 17S)) were significantly enriched in the UA group (variable importance in projection (VIP) > 1, *p* < 0.05) (Figure 4A). Kyoto Encyclopedia of Genes and Genomes (KEGG) enrichment analysis based on the 16 differential metabolites showed that three pathways were significantly enriched. It includes D-amino acid metabolism, arginine biosynthesis, and biosynthesis of various other secondary metabolites (Benjamini–Hochberg false discovery rate (FDR) < 0.05) (Figure 4B).

### 2.5. UA Treatment Alters the Gut Microbiota and Its Metabolic Characteristics and Affects the Host Phenotype

Subsequently, we evaluated the correlation among gut microbiota, serum metabolites, and host phenotype to further explore the possible regulatory mechanisms between gut microbiota characteristics and host phenotype following UA treatment. As indicated by spearman’s correlation coefficient, the four bacteria identified as marker bacteria were significantly correlated with 13 differential metabolites. These metabolites were further associated with BW, TNF-*α*, IL-6, IL-1*β*, IL-10, and TGF-*β* (representing the physiological features of mice) (|R| > 0.6, *p* < 0.05) (Figure 4C). *Odoribacter*, *Coriobacteriaceae_UCG-002,* and *Parabacteroides*, which were identified as the marker bacteria of the UA group, were positively correlated with metabolites (lactucin, ornithine, PA(24:0/22:6(5Z,8E,10Z,13Z,15E,19Z)-2OH(7S, 17S), prolylglycine, and wyerone) enriched in the UA group and were negatively correlated with Indole carboxylic acid sulfate enriched in the CON group. *Romboutsia*, which was identified as the marker bacteria in the CON group, was negatively correlated with 5-hydroperoxypent-1-enylbenzene, Asn Asn Leu Asn Val, dihydroconiferyl alcohol, neoporrigenin B, ornithine, and PA(24:0/22:6(5Z,8E,10Z,13Z,15E,19Z)-2OH(7S, 17S). Prolylglycin and Xi-4-hydroxy-4-methyl-2-cyclohexen-1-one) enriched in the UA group were positively correlated with indole carboxylic acid sulfate enriched in the CON group. Lactucin was negatively correlated with IL-6 and IL-1*β* and positively correlated with BW. Ornithine was negatively correlated with TNF-*α*, IL-6, and IL-1*β*, and positively correlated with BW and IL-10. In addition, there was a direct correlation between the differential bacteria and the host phenotype. *Odoribacter* and *Parabacteroides* were significantly negatively correlated with TNF-*α*, IL-6, and IL-1*β* and significantly positively correlated with BW, TGF-*β*, and IL-10 (Figure S3). *Coriobacteriaceae_UCG-002* was negatively correlated with IL-6 and IL-1*β* (*p* < 0.05) but positively correlated with IL-10 (*p* < 0.05). *Romboutsia* was significantly positively correlated with IL-6 and negatively correlated with IL-10.

### 2.6. UA Alters the Liver Transcriptome

**Figure 5:**
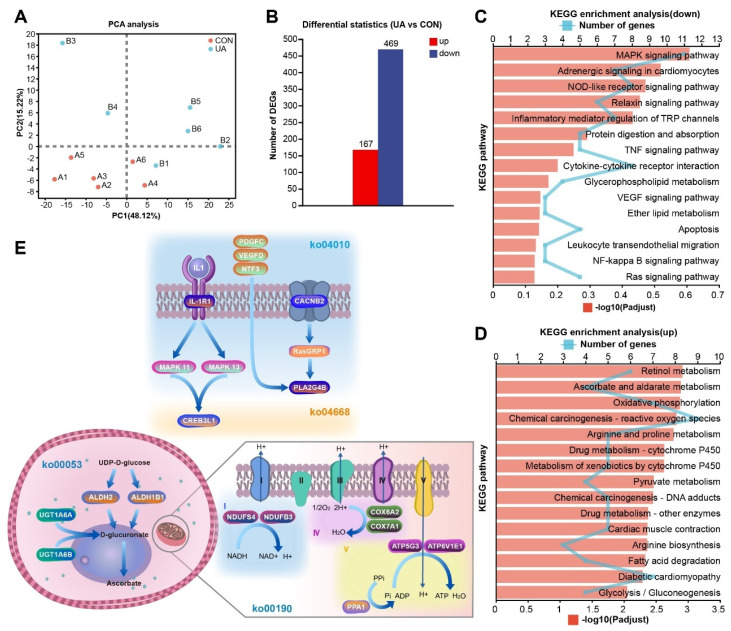
Effect of ursolic acid on the liver transcriptome of mice. (**A**) PCA analysis. (**B**) The number of differentially expressed genes (DEGs). KEGG enrichment pathways of down-regulated (**C**) and up-regulated (**D**) DEGs. (**E**) DEGs related to enrichment pathways (ko04010, ko04668, ko00053, and ko00190). ko04010, MAPK signaling pathway; ko04668, TNF signaling pathway; ko00053, Ascorbate and aldarate metabolism; and ko00190, Oxidative phosphorylation; I, Nicotinamide adenine dinucleotide (NADH) dehydrogenase; II, Succinate dehydrogenase; III, Cytochrome C Reductase; IV, Cytochrome c oxidase; V, ATP synthase. *n* = 6.

To investigate the effects of UA on the mouse liver, we conducted RNA-seq analysis. A total of 29,094 genes were identified in the mouse liver. The results of the PCA analysis indicated a clear separation between the two groups of samples. The first principal component explained 48.12% of the variance between the two groups, and the second principal component explained 15.22% of the variance (Figure 5A). According to the filter threshold |log2FC| ≥ 1 and FDR < 0.05, we identified 636 differentially expressed genes (DEGs) in the UA vs. CON group. Compared to the CON group, there were 167 up-regulated DEGs and 469 down-regulated DEGs in the UA group (Figure 5B). In addition, the expression results of eight random mRNAs detected by qRT-PCR are presented (Figure S4A). The results of RNA-Seq (log2 fold change) and qRT-PCR (log2 fold change) were significantly positively correlated (correlation value = 0.84, *p* < 0.01) (Figure S4B). This indicates the high accuracy of both the sequencing and differential expression analysis results. Gene Ontology (GO) enrichment analysis was performed separately for down-regulated and up-regulated DEGs, and the results showed that the down-regulated genes were significantly enriched to 47 GO terms, such as cell projection organization, chemical synaptic transmission, anterograde trans-synaptic signaling, neuromuscular junction development, centrosome, and triplet codon-amino acid adaptor activity (Figure S5A). The up-regulated genes were significantly enriched in 191 GO terms, including myofilament, skeletal muscle thin filament assembly, and FATZ binding (Figure S5B).

The up-regulated and down-regulated genes were further subjected to KEGG pathway analysis to determine the functional categories. The results showed that there were no significantly enriched pathways among the down-regulated genes, but the pathway with the most enriched genes was the MAPK signaling pathway (DEGs = 11, FDR > 0.05) (Figure 5C). The top five significantly enriched pathways of up-regulated genes were retinol metabolism (DEGs = 6), ascorbate and aldarate metabolism (DEGs = 4), oxidative phosphorylation (DEGs = 7), chemical carcinogenesis-reactive oxygen species (DEGs = 9), and arginine and proline metabolism (DEGs = 5) (FDR < 0.01) (Figure 5D). Based on all DEGs, we further constructed the regulatory mechanism of UA on the ko04010, ko04668, ko00053, and ko00190 pathways (Figure 5E). Interestingly, all DEGs involved in the ko04010 and ko04668 pathways were down-regulated genes (Figure S6A), and all DEGs involved in the ko00053 (Figure S6B) and ko00190 (Figure S6C) pathways were up-regulated genes.

### 2.7. Multi-Omics Integrated Analysis Reveals the Possible Regulatory Mechanism of UA

**Figure 6:**
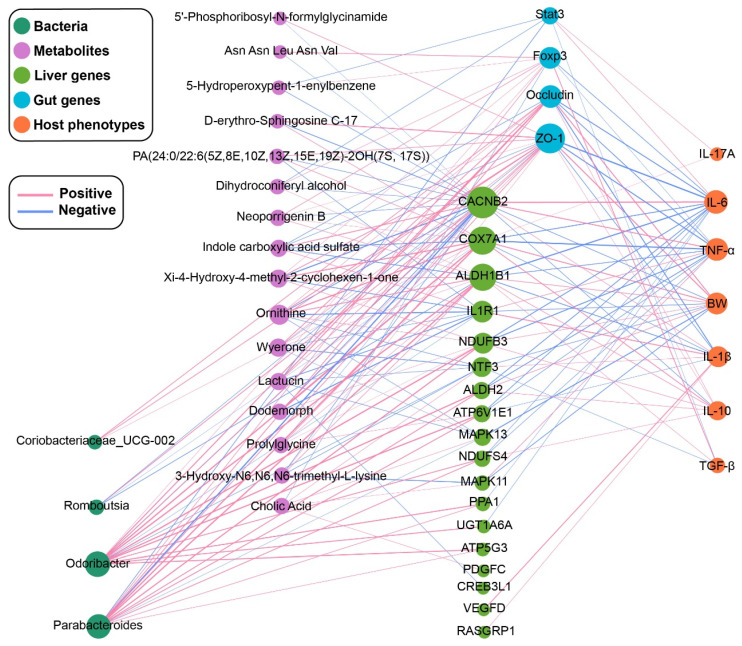
Spearman correlation network among the bacteria, metabolites, liver genes, gut genes, and host phenotypes (|R| > 0.6, *p* < 0.05). The width of the edges is proportional to the correlation strength. The color of the edges: red, positive; blue, and negative, *n* = 6.

Connections between bacteria, metabolites, liver genes, gut genes, and host phenotypes were established through an association study (Spearman correlation coefficient, |R| > 0.6, *p* < 0.05) (Figure 6). BW was positively correlated with intestinal barrier-related genes (*Occludin* and *ZO-1*) and negatively correlated with inflammation-related genes (Mitogen-activated protein kinase (*MAPK)13*, *MAPK11*, Interleukin-1 receptor 1 (*IL1R1*), Neurotrophic factor-3 (*NTF3*), and Calcium (Ca^2+^) voltage-gated channel auxiliary subunit beta 2 (*CACNB2*)). The immune-related cytokines (IL-1*β*, IL-6, and TNF-*α*) were negatively correlated with the intestinal barrier-related genes (*Occludin* and *ZO-1*), and all of them were positively correlated with the inflammation-related gene (*CACNB2*). In addition, IL-1*β* and TNF-*α* exhibited a strong positive correlation with *IL1R1*, of which IL-1*β* had the strongest correlation with *IL1R1* (R = −0.799). *Stat3*, an immune balance-related gene, inhibited the expression of TGF-*β* and promoted the expression of IL-17A, while *Foxp3* facilitated the expression of TGF-*β*. *Odoribacter* and *Parabacteroides* were positively correlated with *Foxp3* and *Parabacteroides* was negatively correlated with *Stat3*. *Coriobacteriaceae_UCG-002*, *Odoribacter*, *Parabacteroides*, wyerone, ornithine, and lactucin could enhance intestinal barrier function by promoting the expression of *Occludin* and *ZO-1*. *Odoribacter*, wyerone, ornithine, and dodemorph showed significant negative correlations with *IL1R1* and *CACNB2*. Notably, *Odoribacter* (16 correlations: 3 negative and 13 positive), *Parabacteroides* (15 correlations: 3 negative and 12 positive), ornithine (10 correlations: 4 negative and 6 positive), wyerone (8 correlations: 4 negative and 4 positive), and lactucin (7 correlations: 4 negative and 3 positive) had the highest number of correlations, suggesting that they may serve as crucial connections in the anti-inflammatory pathway of UA.

## 3. Discussion

UA has garnered considerable attention for its array of pharmacological benefits [6,15,18,19]. In this study, the significant effects of UA in the jejunum were observed, including a notable increase in the V/C ratio and enhancement of intestinal barrier function. These findings highlight UA’s role in promoting digestion and absorption as well as in improving gut health in mice. Th17 and Treg cells, which differentiate from CD4^+^ T cells, are pivotal in the immune response and synergistically contribute to intestinal immune function [20]. The balance between Th17 and Treg cells is crucial for maintaining intestinal immune homeostasis [21]. Disruption of this balance can adversely affect nutrient digestion and absorption, thus compromising overall health [1,21,22].

IL-6-mediated Stat3 signaling stimulates the differentiation of Th17 cells by inducing the nuclear receptor ROR*γ*t, leading to the production of the inflammatory cytokine IL-17A [23]. Subsequently, IL-17A can enhance the secretion of other pro-inflammatory cytokines, including TNF-*α*, IL-6, and IL-1*β* [24]. Conversely, TGF-*β* promotes the transcription of Foxp3, facilitating the differentiation of Treg cells and the production of anti-inflammatory cytokines such as IL-10 and TGF-*β* [25]. Moreover, Foxp3 can inhibit the differentiation of Th17 cells by restricting their transcriptional potential [23]. Our results indicate that UA treatment resulted in the down-regulation of *Stat3* mRNA expression, up-regulation of *Foxp3* mRNA expression, and increased levels of TGF-*β* and IL-10 in mice. These findings suggest that UA can improve intestinal immune homeostasis.

The gut microbiota serves as a critical link between environmental factors and host health [26,27]. Investigating whether UA’s functional role is connected to intestinal microbiota composition has become a topic of interest [13,15,28]. In our study, UA treatment significantly altered the composition and metabolites of the gut microbiota. Notably, the abundance of *Odoribacter* and *Parabacteroides* was significantly increased in the UA-treated mice. *Odoribacter*, a potential probiotic, is known to enhance mucosal immunity and maintain intestinal homeostasis [29]. Its colonization can augment Foxp3/ROR*γ*t regulatory T cells (Treg cells) and induce IL-10 production, thereby mitigating colitis in mice [30,31]. Numerous studies have highlighted the immunomodulatory effects of *Parabacteroides* [32,33,34], which have also been shown to promote CD4^+^ T cell differentiation toward the CD4/Foxp3/IL-10 regulatory pathway [35,36]. Our correlation analysis demonstrated associations between *Odoribacter*, *Parabacteroides*, and key immune factors, including *Foxp3*, *Occludin*, *ZO-1*, IL-1*β*, IL-6, TNF-*α*, and IL-10. These observations suggest that UA’s effects on immune balance and intestinal barrier function may be mediated by *Odoribacter* and *Parabacteroides*.

Furthermore, gut microbiota-derived ornithine has been shown to stimulate ROR*γ*t (+) IL-22 (+) cells and promote gut mucus formation, playing a vital role in maintaining intestinal homeostasis [37]. Previous research has demonstrated that lactucin can modulate gut microbiota and restore intestinal barrier function in inflammatory rat models [38]. Our findings revealed that both ornithine and lactucin were significantly enriched in UA-treated mice, indicating that these metabolites may be key factors in UA-mediated immune balance and intestinal barrier enhancement.

Additionally, our study investigates the effects of UA on liver health in mice. The MAPK signaling pathway is integral to regulating immune responses and plays a critical role in innate immunity [39]. Genes such as *IL1R1*, *MAPK11*, and *MAPK13*, expressed by immune or inflammatory cells, are crucial for inflammatory signaling [40,41]. Ras-guanine nucleotide-releasing protein 1 (RasGRP1) serves as a key regulator of the inflammatory response and is implicated in various inflammatory diseases [42]. Calcium (Ca^2+^) voltage-gated channel auxiliary subunit beta 2 (CACNB2) may contribute to aberrant RAS-MAPK activation [43]. Our study found that *IL1R1*, *MAPK11*, *MAPK13*, *CACNB2*, and *RasGRP1* expression levels were significantly downregulated in the livers of mice in the UA group, suggesting that UA may lower the risk of liver inflammation and improve liver homeostasis. Moreover, the upregulated genes in the UA group were significantly enriched in pathways related to retinol metabolism, ascorbic acid and aldarate metabolism, and oxidative phosphorylation. Retinol, a form of vitamin A, plays an unexpected but crucial role in regulating immune responses [44]. Ascorbate, or vitamin C, acts as a potent antioxidant that supports various immune functions and enhances immune defenses [45]. Mitochondrial oxidative phosphorylation is responsible for generating the majority of ATP, which is essential for sustaining life and maintaining metabolic homeostasis [46]. These results imply that UA may enhance liver health by promoting the synthesis of retinol and ascorbic acid, thereby regulating metabolic homeostasis in the liver.

Interestingly, we observed that the *IL1R1* and *CACNB2* genes, which are associated with the MAPK signaling pathway, are broadly linked to immune-related factors, bacteria (*Odoribacter* and *Parabacteroides*), and metabolites (ornithine and lactucin). *Parabacteroides distasonis* has been shown to reduce liver damage and inflammation by inhibiting the NF-κB/MAPK pathway and activating the Nrf2 pathway, which may be related to the improvement in amino acid and bile acid metabolism in intestinal microorganisms [47]. *Parabacteroides distasonis* has a protective effect against non-alcoholic steatohepatitis (NASH) in mice by producing beneficial metabolites from dietary fiber [48]. In addition, *Odoribacter* has also been shown to be significantly negatively associated with measures of NASH progression [49]. Previous studies suggest that the liver-protective mechanism of ornithine may involve its conversion into the antioxidant glutathione and the nitric oxide precursor L-arginine, both of which are crucial for improving liver microcirculation [50,51]. Additionally, lactucin has been shown to downregulate the MAPK pathway, further enhancing liver health [52,53]. Lactucin may also enhance GLA circulation and reduce liver inflammation [38]. These observations suggest that the effects of UA on liver health may be mediated by regulating gut microbiome composition and its metabolism. However, our correlation analysis offers only indicative and predictive insights and does not definitively establish causal relationships between metabolites and hepatoprotective effects. Further studies are required to validate the relationship between these metabolites and liver health.

Our data can help us understand the function of UA in regulating immune homeostasis and improving liver health and identify the key role that gut microbiota and its metabolites may play in this process. However, we acknowledge that some limitations should be noted. We did not assess changes in gut microbial function and its association with inflammation in UA-treated mice. Further systematic studies, such as metagenomic, metatranscriptomic, or other multi-omics investigations, are needed to identify the function of gut microbiota and further explain the impact of changes in microbial metabolic function on inflammation.

In conclusion, UA can improve immune balance and enhance intestinal barrier function in mice. UA is able to increase beneficial bacteria (*Odoribacter* and *Parabacteroides*) and their metabolites (ornithin and lactucin). UA can inhibit the expression of *IL1R1* and *CACNB2* mRNA in the liver, upregulate the synthesis pathway of retinol and ascorbic acid in the liver, and promote liver health.

## 4. Materials and Methods

### 4.1. Ethics Statement

All experiments were performed in accordance with the ARRIVE guidelines and were carried out in accordance with the U.K. Animals (Scientific Procedures) Act, 1986 and associated guidelines, EU Directive 2010/63/EU for animal experiments, and approved by the Ethics Committee of Animal Experimentation of Hebei Agricultural University (Protocol 2023058).

### 4.2. Animals and Experimental Design

Eight-week-old female C57BL/6J mice were purchased from SPF Biotechnology Co., Ltd. (Beijing, China) and housed in pathogen-free facilities with a 12-h light and 12-h dark cycle at 22 °C.

After acclimatization to the laboratory conditions for 1 week, mice were randomly divided into 5 groups (*n* = 6 per group), including the control (CON) group, the UA 5, UA 25, UA 100, and UA 250 groups. Mice in the CON group were provided with a daily oral administration of 100 μL of PBS. Mice from the UA 5, UA 25, UA 100, and UA 250 groups received 5, 25, 100, and 250 mg/kg body weight of UA (purity ≥ 98%, Shenniu Pharmaceutical Co., Ltd., Dezhou, China; Figure S7) added to 100 μL PBS by daily gavage for 21 days. All group mice were administered menstruum, which was isovolumetric with 0.5% sodium carboxymethyl cellulose and 1% Tween 80. UA was dissolved in that menstruum (the menstruum and UA were administered directly into the stomach using an oral gavage needle).

Body weight was measured every three days throughout the study. On the 21st day, the mice were anesthetized with ether, the eyeballs were removed, blood samples were taken in 1.5 mL centrifuge tubes, and serum was separated by centrifugation for 15 min (3500× *g*, 4 °C), after which the mice were sacrificed. Fecal samples from all mice were collected aseptically and stored at −80 °C for microbiome and metabolomic analyses. The middle segments (0.5 cm) of the duodenum, jejunum, and ileum were flushed with PBS and fixed in a 10% formaldehyde solution for histological observation. Jejunum and liver tissue were washed with PBS, cut into slices, snap-frozen in liquid nitrogen, and stored at −80 °C for future analysis.

### 4.3. Enzyme-Linked Immunosorbent Assay (ELISA)

Serum samples were measured for the levels of IL-1*β* (MM-0040M2), IL-6 (MM-0163M2), TNF-*α* (MM-0132M2), TGF-*β* (MM-0689M2), IL-10 (MM-0176M2), and IL-17A (MM-0759M2) according to the instructions of specific ELISA kits (Jiangsu Meimian Industrial Co., Ltd., Yancheng, China).

### 4.4. Histological Analysis

The fixed duodenum, jejunum, and ileum samples were embedded in paraffin and stained with hematoxylin–eosin to obtain cross-sectional sections. In each section, intact and representative villi were selected, and intestinal morphology was measured. The images were evaluated using the ImageJ software 1.53j (US National Institutes of Health, Bethesda, MD, USA). Villus height (VH), crypt depth (CD), and intestinal wall thickness were measured. The ratio of villus height to crypt depth (V/C) was calculated.

### 4.5. Quantitative Reverse Transcription-Polymerase Chain Reaction (qRT-PCR)

Total RNA extraction and quantitative real-time PCR were performed as described previously. The extracted RNA was dissolved in RNase-free water and quantified using a NanoDrop-2000 spectrophotometer (Thermo Fisher Scientific, Waltham, MA, USA). RNA purity was evaluated by measuring the absorbance ratio at 260:280 nm, while RNA integrity was confirmed by the detection of the 18S and 28S bands after electrophoresis in 1% agarose gels. The primer sequences are shown in Table S1. The standard curve was drawn for each gene, and the linear relationship and amplification efficiency were calculated. Primers were confirmed as follows: correlation coefficient (R^2^) > 0.98; the slope of the standard curve: −3–−3.5; and the amplification efficiency: 0.9–1.2. The mRNA levels of target genes were normalized to *β-actin* mRNA. Based on the cycle threshold (CT) value, the *β-actin* mRNA level was stable across the treatments in this study (*p* > 0.1) (Figure S8). The results of the relative mRNA expression of genes were calculated using the 2^−ΔΔCT^ method [54].

### 4.6. 16S rRNA Gene Sequencing

Microbial genomic DNA was isolated from fecal specimens utilizing the E.Z.N.A.^®^ soil DNA kit (Omega Bio-tek, Norcross, GA, USA). The V3-V4 hypervariable region of the bacterial 16S rRNA gene underwent amplification with the primer combinations 338F (5’-ACTCCTACGGGAGGCAGCAG-3’) and 806R (5’-GGACTACHVGGGTWTCTAAT-3’) via the T100 Thermal Cycler PCR thermocycler (BIO-RAD, Hercules, CA, USA). Subsequently, the purified amplicons were combined in equimolar proportions and subjected to paired-end sequencing on an Illumina PE300 platform (Illumina, San Diego, CA, USA) in accordance with standard operating procedures established by Majorbio Bio-Pharm Technology Co., Ltd. (Shanghai, China).

### 4.7. Microbiota Data Analysis

Initial sequencing data underwent quality filtering using Fastp version 0.19.6, followed by read merging using FLASH version 1.2.11. Subsequently, the sequences were optimized and grouped into operational taxonomic units (OTUs) at a 97% sequence similarity threshold utilizing UPARSE 11. The taxonomic classification of each OTU representative sequence was conducted, employing RDP Classifier version 2.13 against the Silva v138 16S rRNA gene database, with a confidence threshold set at 0.7.

Analysis of fecal microbiota data was executed on the Majorbio Cloud platform (https://cloud.majorbio.com (accessed on 15 April 2024)). Rarefaction curves and alpha diversity indices were computed using Mothur v1.30.1. Principal coordinate analysis (PCoA) and Adonis analysis, based on Bray–Curtis dissimilarity, were employed to assess the similarity among microbial communities across different samples, facilitated by the “Vegan” package. The statistical significance of non-parametric relative abundance profiles was evaluated utilizing the Mann–Whitney test. Additionally, linear discriminant analysis (LDA) effect size (LEfSe) analysis was conducted to identify significantly enriched bacterial taxa among distinct groups (LDA score > 3, *p* < 0.05).

### 4.8. Liquid Chromatograph–Mass Spectrometer Analysis (LC–MS Analysis)

A solid sample weighing 50 mg underwent metabolite extraction utilizing 400 μL of a 4:1 (*v*:*v*) methanol–water extract solution. LC–MS/MS analysis was performed on a SCIEX UPLC-Triple TOF 6600 system featuring an ACQUITY HSS T3 column (100 mm × 2.1 mm i.d., 1.8 μm; Waters, Milford, MA, USA) at Majorbio Bio-Pharm Technology Co., Ltd. (Shanghai, China). Mobile phase A is 95% water + 5% acetonitrile (containing 0.1% formic acid); mobile phase B is 47.5% acetonitrile + 47.5% isopropyl alcohol + 5% water (containing 0.1% formic acid). The flow rate was 0.40 mL/min, the sample size was 10 μL, and the column temperature was 45 °C. Positive ion mode separation gradient 0–3 min and mobile phase B increased from 0% to 20%; in 3–4.5 min, mobile phase B increased from 20% to 35%; in 4.5–5 min, mobile phase B increased from 35% to 100%; at 5–6.3 min, mobile phase B remained 100%; and in 6.3–6.4 min, mobile phase B decreased from 100% to 0%. At 6.4–8 min, mobile phase B maintained 0%. Negative ion mode separation gradient: 0–1.5 min, mobile phase B from 0 to 5%; 1.5–2 min, mobile phase B increased from 5% to 10%; 2–4.5 min, mobile phase B increased from 10% to 30%; 4.5–5 min, mobile phase B increased from 30% to 100%; and at 5–6.3 min, the linearity of mobile phase B remained 100%. In 6.3–6.4 min, mobile phase B decreases from 100% to 0%. The linearity of mobile phase B remained at 0% from 6.4–8 min. Positive and negative ion scanning modes are used to collect the sample quality spectrum signal, and the quality scanning range is 50–1200 m/z. The spray gas flow rate is 50 psi, the auxiliary gas flow rate is 50 psi, the curtain gas flow rate is 35 psi, the ion source temperature is 500 °C, the positive mode ion spray voltage is set to 5500 V, the negative mode ion spray voltage is set to −4500 V, and the ion transfer tube temperature is 325 °C. Normalized collision energy is 20-40-60 eV cyclic collision energy. DDA mode was used to collect data.

Raw UHPLC–MS data were processed using Progenesis QI 2.3 software (Nonlinear Dynamics, Waters, USA) for peak detection and alignment. Metabolic features detected in at least 80% of samples were retained, and minimum imputation was applied to samples falling below the quantitation limit. The normalization of each metabolic feature was conducted by sum and quality control. Variables with relative standard deviation (RSD) exceeding 30% of QC samples were excluded, and log10 transformation was performed to obtain the final data matrix.

Conduct variance analysis on the processed data matrix file. The “ropls” R package (Version 1.6.2) was utilized for partial least squares discriminant analysis (PLS-DA). Metabolites with a variable importance in projection (VIP) value exceeding 1 and a significance level of *p* < 0.05 were deemed significantly different. Differential metabolites between the two groups were subjected to metabolic enrichment and pathway analysis based on the Kyoto Encyclopedia of Genes and Genomes (KEGG) database (http://www.genome.jp/kegg/ (accessed on 15 April 2024)) to map their biochemical pathways.

### 4.9. RNA Extraction, Library Construction, and Sequencing

Total RNA was isolated from the tissue using TRIzol^®^ Reagent as per the manufacturer’s protocol. Subsequently, RNA integrity was assessed using a 5300 Bioanalyser (Agilent, Santa Clara, CA, USA) and quantification was carried out using the ND-2000 (NanoDrop Technologies, Wilmington, DE, USA). RNA purification, reverse transcription, library preparation, and sequencing procedures were conducted at Shanghai Majorbio Bio-pharm Biotechnology Co., Ltd. (Shanghai, China) following the guidelines provided by Illumina (San Diego, CA, USA). The RNA-seq transcriptome library was constructed using Illumina^®^ Stranded mRNA Prep and Ligation Kit from Illumina (San Diego, CA, USA), utilizing 1 μg of total RNA. Libraries underwent size selection for cDNA fragments of 300 bp on 2% low-range ultra-agarose, followed by PCR amplification with Phusion DNA polymerase (NEB) for 15 PCR cycles. Following quantification with Qubit 4.0, paired-end RNA-seq libraries were sequenced using the NovaSeq 6000 sequencer (Illumina, San Diego, CA, USA) (2 × 150 bp read length).

The raw paired-end reads underwent trimming and quality control using Fastp with default parameters. Subsequently, clean reads were individually aligned to the reference genome in orientation mode employing HISAT2 software 2.2.1. The mapped reads of each sample were assembled by StringTie v2.1.4 utilizing a reference-based approach.

To identify differential expression genes (DEGs) between distinct samples, the expression level of each transcript was quantified utilizing the transcripts per million reads (TPM) method via RSEM. Differential expression analysis was conducted using DESeq2 (version 1.12.3), with DEGs meeting the criteria of |log2FC| ≥ 1 and false discovery rate (FDR) < 0.05 considered significantly differentially expressed genes. Additionally, functional enrichment analysis, encompassing Gene Ontology (GO) and KEGG pathways, was performed to discern which DEGs were enriched in GO terms and metabolic pathways.

### 4.10. Correlation Analysis

Given the extensive diversity, dimensional complexity, and discontinuous distribution of omics data, we applied the Spearman correlation examination in this investigation to investigate the associations among the bacteria, metabolites, liver genes, gut genes, and host phenotypes. A threshold of |R| > 0.6, with *p* < 0.05, was utilized to discern noteworthy correlations. Cytoscape version 3.9.0 was employed for network construction. Visualization of the interrelations between microbiome, metabolome, and physiological attributes was accomplished utilizing the R package “ggplot2”.

### 4.11. Statistical Analysis

The body weight, ELISA, and histological observation data were analyzed and one-way analysis of variance (ANOVA) by Statistical Package for the Social Sciences (SPSS) version 26; the differences between means were compared by Duncan’s multiple range test. Gene relative expression was compared using the *t*-test. The results were expressed as mean ± standard deviation (SD). The significance was set at *p* < 0.05.

## Data Availability

The data presented in this study are openly available in the NCBI Sequence Read Archive (SRA), reference number PRJNA1115940.

## Supplementary Materials

The supporting information can be downloaded at https://www.mdpi.com/article/10.3390/ijms251910623/s1.
