# Supplementary material for: Ursolic Acid Regulates Immune Balance, Modulates Gut Microbial Metabolism, and Improves Liver Health in Mice
Source: Int J Mol Sci. 2024 Oct 2;25(19):10623. doi: 10.3390/ijms251910623 (PMC11477038; doi:10.3390/ijms251910623)
Supplement: Supplementary file 1 [file ijms-25-10623-s001.zip › ijms-3188492-supplementary.pdf]

Supplemental Tables and Figures

Table S1. Gene-specific primer sequences used for gene transcription analyses.

Gene	Genebank accession number	Primers sequences (5'→3')	Product size (bp)
β-actin	NM_007393.5	F: CGTTGACATCCGTAAAGACC<br>R: AACAGTCCGCCTAGAAGCAC	281
Foxp3	NM_001199347.1	F: GGGATCAATGTGGCCAGTCT<br>R: CAGCCAAAAGGTTGCTGTCTT	103
RORγt	NM_001293734.1	F: CTCATCAATGCCAACCGTCC<br>R: GCTAGGAGGCCTTGTCGATG	119
Stat3	NM_213659.3	F: GCAATACCATTGACCTGCCG<br>R: AACGTGAGCGACTCAAACTG	115
Occludin	NM_001360536.1	F: CAGACCTGATGAATTCAAACCCA<br>R: AGAGTACGCTGGCTGAGAGA	100
Claudin-1	NM_016674.4	F: TGTGTCCACCATTGGCATGA<br>R: ACTAATGTCGCCAGACCTGAAA	117
ZO-1	NM_009386.3	F: CTGGTGAAGTCTCGGAAAAATG<br>R: CATCTCTTGCTGCCAAACTATC	97
ASPG	NM_001081169.1	F: GCTATGGGACCAGAGCGGC<br>R: CAGGTACCAACACTCCGCC	85
CEP85	NM_144527.3	F: GTTCTCTGGGAACTGAGTGGC<br>R: CACTTGAGCTGCAAAAATCCTCT	147

ECHDC2	NM_001254754.1	F: GATGAGTGAGATCGCAGCCT<br>R: GCTGCTATTCGGAGGTCACA	108
SELENOP	NM_001042613.2	F: TGACAGTGTGCGGAAAACCT<br>R: GGCTGATTTTGTCAGGCAGC	116
MUP18	NM_001199333.1	F: CAGCTGATGGGGCTCTATGG<br>R: GCAGCGATTGGCATTGGATA	129
MUP8	XM_030253011.2	F: CAGCTGATGGGGCTCTATGG<br>R: GAGGCAGCGATTGGCATTG	132
RBP4	NM_001159487.1	F: AGAGTTTGGCTCCACCGAGA<br>R: CAGAGCCCAGAGAAACGAGC	239
TRF	NM_133977.2	F: TGTGACCTGTGTATTGGCCC<br>R: GGCAGAGCAACTCGAAGTCT	202

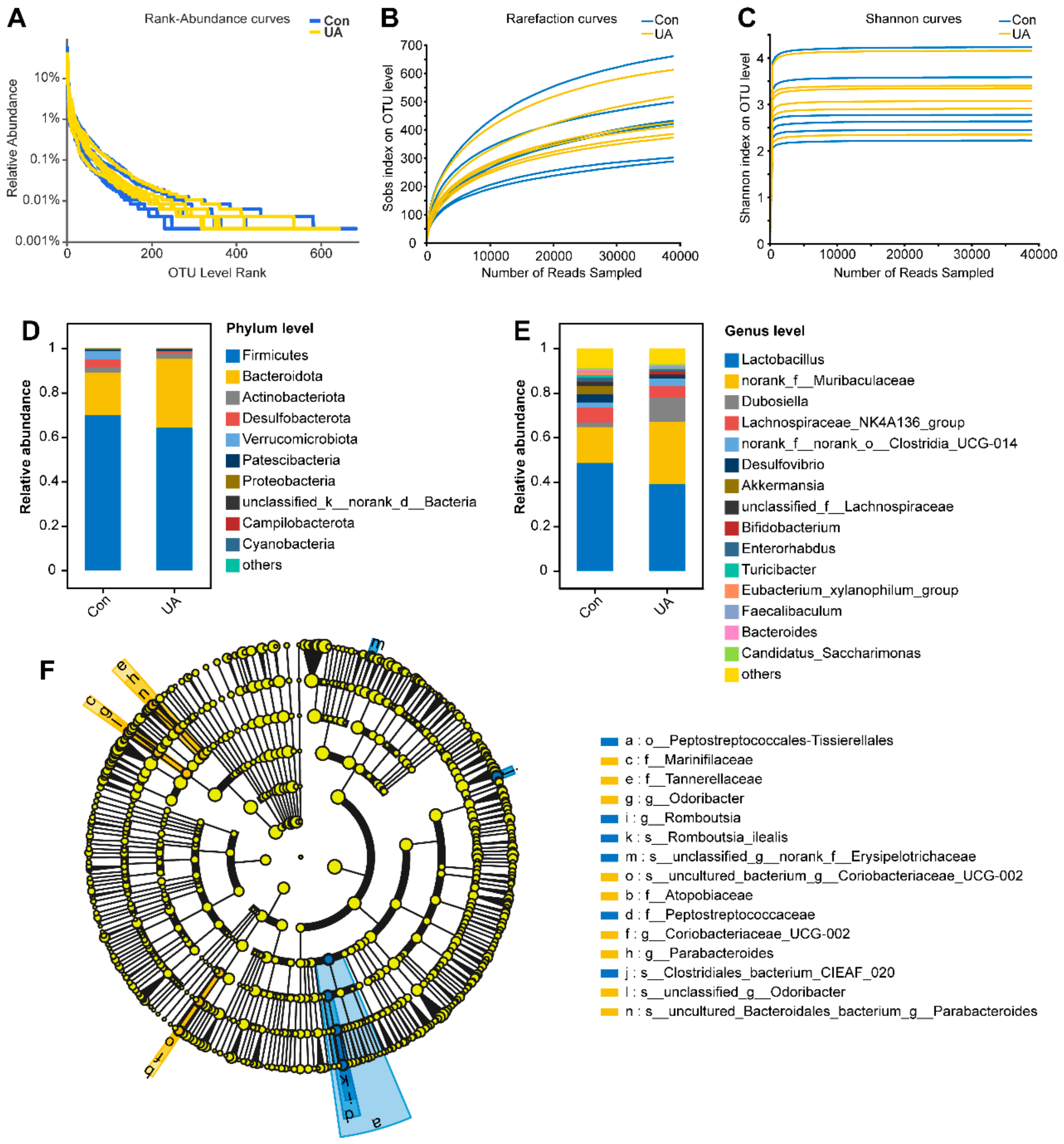

Figure S1. Effect of ursolic acid on gut microbiota in mice. (A) Rank abundance curve. (B) Sobs curve. (C) Shannon curve. The relative abundance of gut bacteria at the (D) phylum and (E) genus levels. (F) Differentiating bacteria identified by LEfSe with LDA effect size ≥ 3. n = 6.

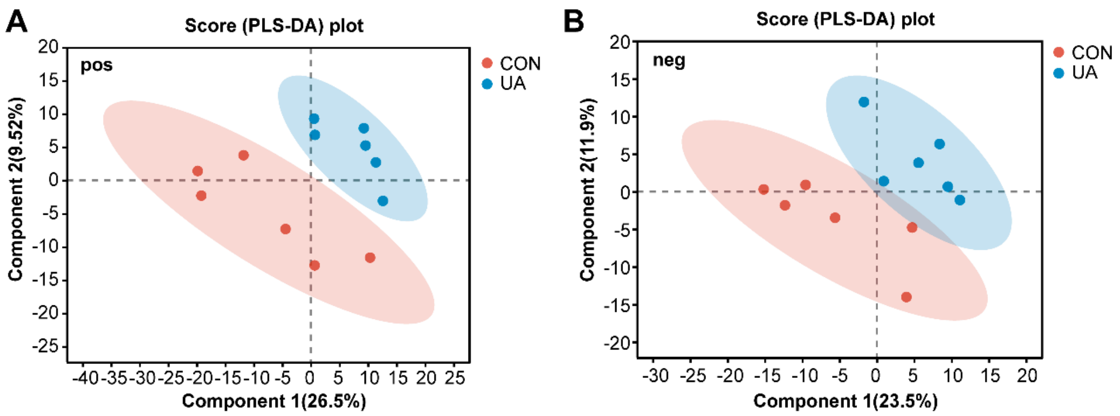

Figure S2. Effect of ursolic acid on the fecal metabolome of mice. Partial Least Squares Discriminant Analysis (PLS-DA) under (A) positive ion mode (pos) and (B) negative ion mode (neg).

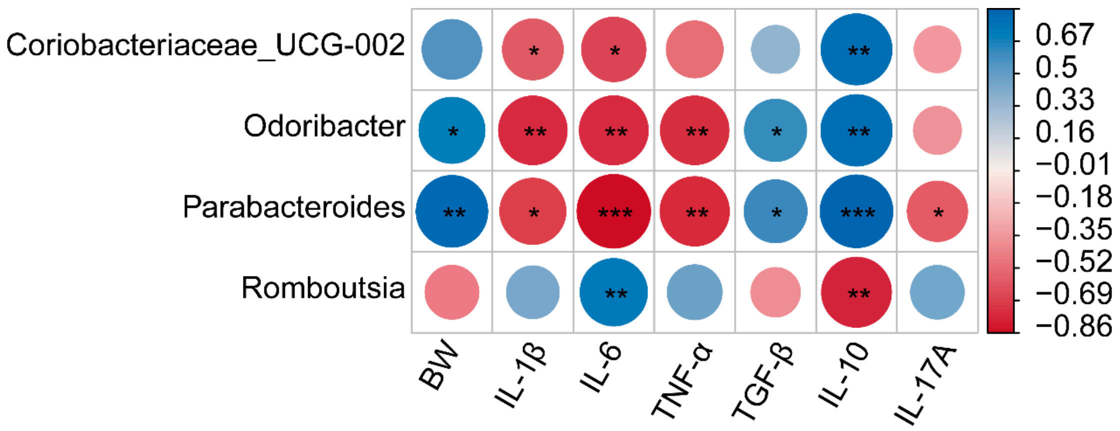

Figure S3. Heat map of correlation between differential bacteria and physiological feature. Red circles indicate a positive correlation (Spearman correlation test, |R| > 0.6), while blue circles show correlations that were negative (Spearman correlation test, |R| > 0.6). A larger circle indicates a indicates a smaller *P*-value (*, *P* < 0.05; **, *P* < 0.01; ***, *P* < 0.001), darker circles indicate larger |R|.

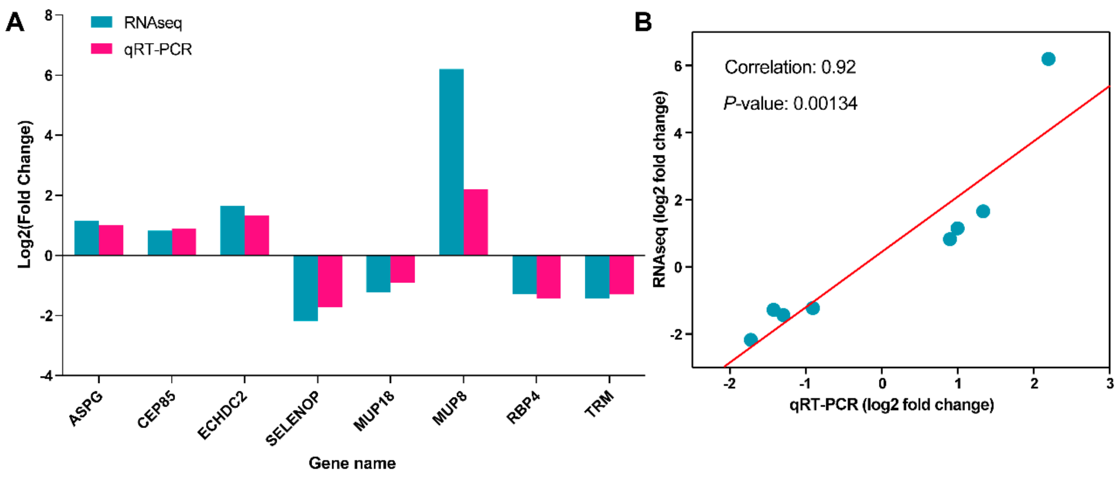

Figure S4. Quantitative validation of liver transcriptome data. (A) Verification of the expression levels of 8 mRNAs using qRT-PCR. (B) Log2 fold change correlation between RNASeq and qRT-PCR.

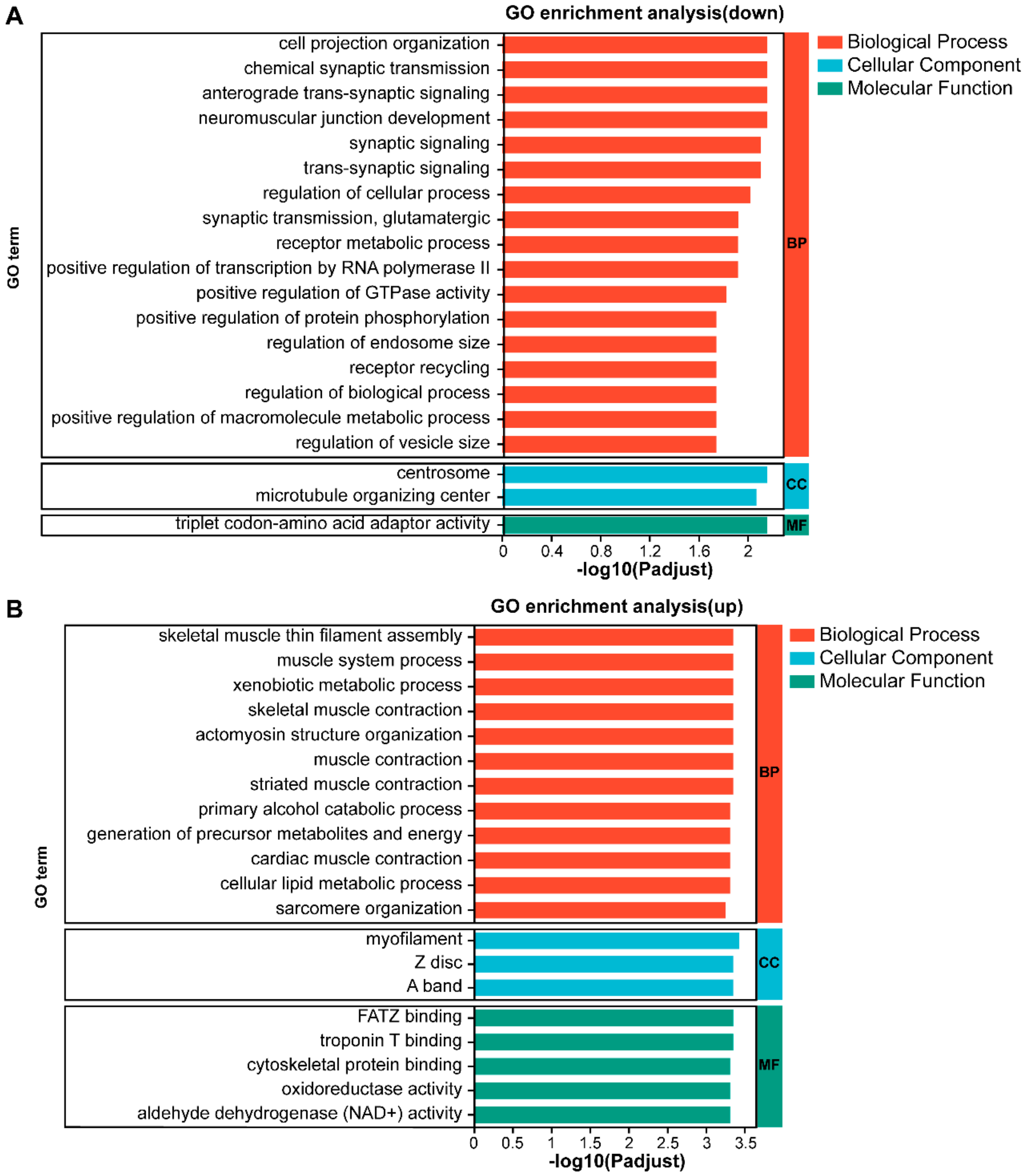

Figure S5. GO enrichment analysis of down-regulated (A) and up-regulated (B) DEGs.

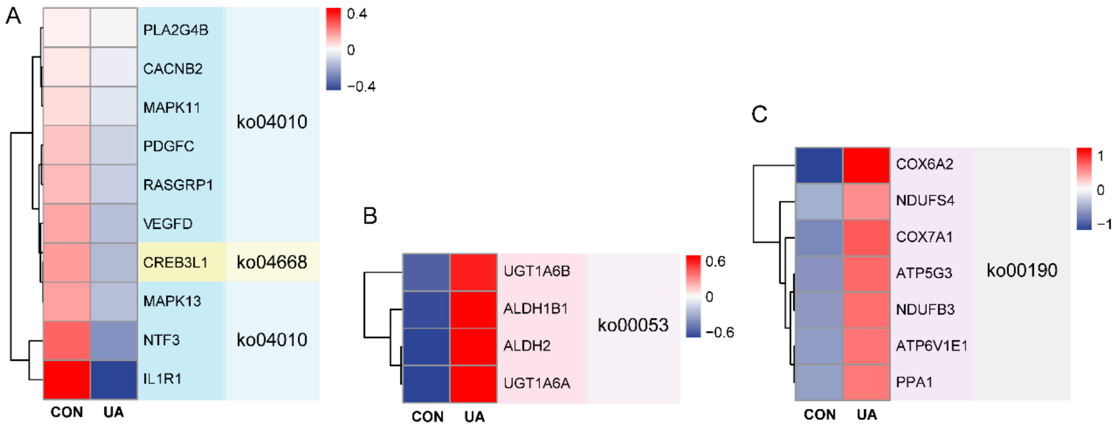

Figure S6. (A-C) Heatmap of DEGs related to enrichment pathways (ko04010, ko04668, ko00053 and ko00190). ko04010, MAPK signaling pathway; ko04668, TNF signaling pathway; ko00053, Ascorbate and aldarate metabolism; and ko00190, Oxidative phosphorylation. n = 6.

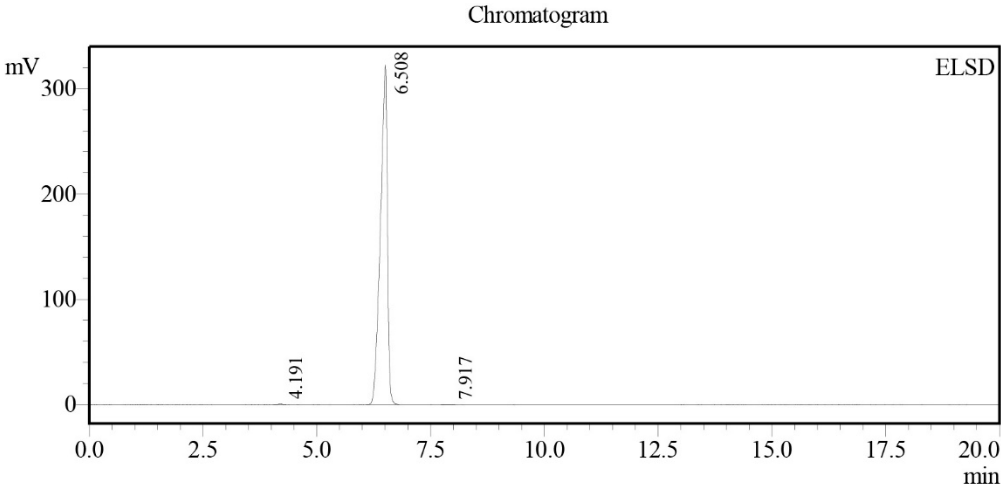

ELSD

PeakTable

Peak#	Ret. Time	Area	Height	Area %	Height %
1	4.191	3978	852	0.116	0.264
2	6.508	3438433	321998	99.862	99.691
3	7.917	785	145	0.023	0.045
Total		3443196	322995	100.000	100.000

Figure S7. Chromatogram of ursolic acid.

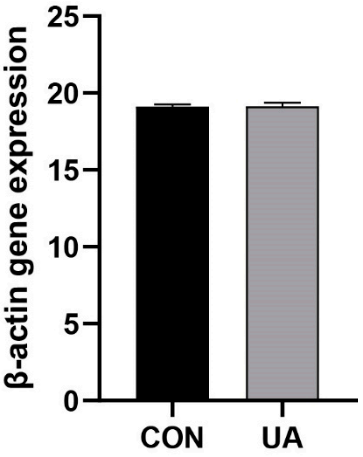

Figure S8. The reference gene (β-actin) in different groups of mice.
